# Threat of Hantavirus Pulmonary Syndrome to Field Biologists Working with Small Mammals

**DOI:** 10.3201/eid1309.070445

**Published:** 2007-09

**Authors:** Douglas A. Kelt, Dirk H. Van Vuren, Mark S. Hafner, Brent J. Danielson, Marcella J. Kelly

**Affiliations:** * University of California, Davis, California, USA; † Louisiana State University, Baton Rouge, Louisiana, USA; ‡ Iowa State University, Ames, Iowa, USA; § Virginia Polytechnic Institute, Blacksburg, Virginia, USA

**Keywords:** Hantavirus pulmonary syndrome, personal protective equipment, field biologists, Sin Nombre virus, synopsis

## Abstract

Low risk for hantavirus pulmonary syndrome (HPS) has been reported among biologists engaged in fieldwork with rodents. The overall probability of acquiring HPS when working with rodents appears to be 1 in 1,412 (0.00071). Nonetheless, a causal link between HPS and lack of personal protective equipment (PPE) use is suggested by some investigators. However, supporting data are incomplete and consequently misleading. A recent HPS case was assumed to be acquired during rodent-handling activities, although substantial peridomestic exposure was evident. Regulatory groups interpret inadequate data as evidence of the need for rigorous PPE, which can hamper field research and instructional efforts. PPE recommendations should be reviewed and revised to match the risk associated with different types of fieldwork with small mammals.

## Abstract 2

Field biologists should use personal protective equipment appropriate for their activities.

Hantavirus pulmonary syndrome (HPS) is an uncommon disease associated primarily with exposure to deer mice (*Peromyscus maniculatus*), widespread rodents that serve as the reservoir host for Sin Nombre virus (SNV) (*1*). The virus is shed in the saliva, urine, and feces of the host, and transmission to humans is thought to result primarily from inhaling infectious, aerosolized saliva or excreta, especially when entering or cleaning rodent-infested structures (*2*,*3*). The virus presumably may also be transmitted by a bite from an infected deer mouse, but this type of transmission is considered rare (*4*). The disease is difficult to treat, especially in advanced stages, and mortality rates are ≈30%–35%; thus, prevention is important (*3*). Development of effective preventive measures requires a logical match between mechanisms of transmission and the various protective devices and precautionary measures that have been advocated as well as accurate assessment of occupational risks for exposure.

Workers who frequently handle wild rodents are presumed to be at greater risk for exposure to SNV (*2*). Thus, in 1994 staff from the Centers for Disease Control and Prevention (CDC) visited several national conferences and took blood samples from field mammalogists whose jobs entailed various levels of direct exposure to small mammals by live and kill trapping. Data for 757 of these donors were recently published in Emerging Infectious Diseases (*5*) and documented that only 4 (0.528%) of 757 active field mammalogists with “a history of exposure to rodents in North America and…of occupational exposure to deer mice …” had positive test results for SNV exposure. The authors concluded (abstract) “that the risk of infection with hantaviruses … is low in persons whose occupations entail close physical contact with … rodents [including deer mice] … in North America.” They also cited 3 other studies of workers in occupations with high risk for exposure to rodents in which no SNV-positive cases were documented from 583 (*6*,*7*) and 72 persons (*8*). Summation of results from these 4 studies indicated that only 4 (0.283%) of 1,412 persons in high-risk occupations had antibodies to SNV.

Although their data indicate that fieldwork with mammals has minimal risk for contracting HPS, Fulhorst et al. (*5*) implied a causal link between infection in the 4 HPS-positive mammalogists and their failure to use personal protective equipment (PPE) while handling rodents in the field (“None of the 4 persons in the study who were antibody-positive against SNV had worn gloves, masks, or protective eyewear when handling rodents …”). Such an implication is unwarranted, given that ≈70% of all persons tested by Fulhorst et al. never (or infrequently) wore any protective equipment while handling rodents in the field. Because most of the testing was done before widespread public awareness of HPS, it is likely that none of the persons tested in 1994 wore protective equipment designed to prevent exposure to hantaviruses in the field.

Only 1 of the 4 SNV-positive persons in the study by Fulhorst et al. reported having been “hospitalized for an illness characterized by fever, headache, and severe shortness of breath (symptoms suggestive of HPS).” The distinction between SNV, the causative agent, and HPS, the manifestation of illness, is important but often overlooked. Whereas 0.528% of samples in the study by Fulhorst et al. had antibodies to SNV, only 0.132% of the persons tested in that study actually exhibited symptoms of HPS. This number decreases to 0.071% (0.00071) if all 1,412 serologic samples (see above) are considered. Thus, in the absence of any data on the proportion of exposed persons (SNV reactive) who become ill with HPS, one could argue that the risk for illness among mammalogists working in the field may be 25% of that reported by Fulhorst et al. Moreover, the single known field mammalogist who contracted HPS (one of the authors, B.J.D.) was living in a mouse-infested building near his field site at the time he was infected.

Fulhorst et al. (*5*) noted that “2 recent HPS cases …underscore the need to use … personal protective equipment and follow recommended safety procedures …” One of these cases was in a field technician who was employed by 2 of us (D.A.K. and D.H.VV.) in a study in the Sierra Nevada of California. Unfortunately, data for this patient were incomplete. Fulhorst et al. (*5*) noted that our employee “was trapping rodents as part of a forest health study in California,” but they did not report documented evidence of extended residential exposure to SNV. The implication of Fulhorst et al. was that HPS in this case was acquired through direct contact with rodents in the field. However, our field crew had been living for 2 months in a seasonal cabin that was inhabited by hantavirus-positive deer mice. Testing by the California Department of Health Services Vector-Borne Disease Section (CA-VBDS) documented serum antibodies to SNV in 2 of 4 deer mice trapped in this cabin (*9*). Field sampling by the CA-VBDS resulted in the capture of 50 deer mice, 16 of which (32%) tested positive for antibodies to SNV. These positive samples were found at only 2 of our 18 field sites (in 1 of 5 and 7 of 14 deer mice, respectively) but at all 3 areas sampled at our field camp (3 of 5, 3 of 17, and 2 of 4 deer mice). Field sites ranged from several kilometers to >30 km from our field camp.

Thus, the evidence in this instance points to 2 potential sources of infection: direct handling of rodents in the field or residential exposure to aerosolized hantavirus particles. All data published regarding SNV indicate that the primary route of exposure is by inhalation of aerosolized viral particles in a peridomestic setting (e.g., [*4*]). We acknowledge that our employee may have acquired HPS by occupational exposure in the field, but the available evidence demonstrates that acquisition by peridomestic exposure was at least equally possible. If one considers that 70% of HPS cases are associated with peridomestic exposure (*10*) and that our employee was sleeping, eating, and even shaking out dusty rugs in a cabin inhabited by SNV-positive rodents, a residential source of infection seems most probable. Unfortunately, studies such as that by Fulhorst et al. (*5*) tend to focus the attention of the public and safety administrators on the potential dangers of field mammalogy (e.g., trapping and handling of rodents in the field) and away from the more likely (peridomestic) source of exposure to hantaviruses. Because regulatory bodies, such as institutional animal care and use committees, often “play it safe” by turning CDC safety recommendations into safety requirements for their constituents, many field mammalogists today are required to wear PPE while handling rodents in the field (with a documented 0.071% probability of acquiring HPS [*5*]) but are allowed to sleep and eat unprotected in field cabins potentially infested with viremic rodents.

We are not calling for relaxation or abolition of PPE. Rather, we are trying to emphasize that PPE should be suitable and appropriate for the occupational risk. Additionally, PPE recommendations should be reconsidered when new data suggest that either additional or reduced levels of PPE are warranted. Field work on mammals that involves virologic or blood sampling or other direct contact with body fluids and organs almost certainly involves greater risk for exposure to SNV than mark-recapture live-trapping studies in which the greatest contact with bodily fluids is with rodent urine or during application of an eartag. Although nitrile or latex gloves are reasonable PPE for protecting skin from urine, use of surgical gowns, shoe covers, and high-efficiency particulate air filter–fitted respirators (all recommended by Mills et al. [*2*]) are likely inappropriate and excessive relative to the risk associated with handling, marking, and releasing small mammals in open-air conditions. Current CDC recommendations do not distinguish between invasive and noninvasive studies. As such, these recommendations for PPE against the entire array of potential risk factors lead to cumbersome and likely ineffective PPE for students learning to live-trap small mammals and field workers wishing merely to apply an eartag and release the animal. For such activities, we believe that available data strongly argue against the PPE recommendations currently provided by CDC.

SNV has been present in North America for a long time (*3*,*11*), and all evidence indicates that it will continue to show cyclic increases and decreases in rodent populations coincident with increases and decreases in rodent density (*11*). This virus is unstable in the presence of sunlight, detergents, bleach, and other agents (*12*); combined data of Vitek et al. (*6*), Zeitz et al. (*7*), Fritz et al. (*8*), and Fulhorst et al. (*5*) document that HPS is an uncommon disease that is difficult to acquire by handling rodents in the field. Although the mortality rate for persons hospitalized with HPS remains high, awareness of HPS symptoms and treatments of the disease are improving, as is prognosis for recovery if the disease is diagnosed promptly.

Fulhorst et al. (*5*) document that field research in ecology and biology of small mammals, including deer mice, poses an extremely low risk for field workers. However, efficacy of protective equipment in reducing exposure to SNV in the field remains unknown. We call for increased objectivity in future studies of HPS risk, especially with regard to possible sources of infection. Open communication between field biologists involved in HPS cases, CDC, and healthcare professionals investigating these cases would be in the best interest of all parties.
